# Supplementary material for: MicroSEC filters sequence errors for formalin-fixed and paraffin-embedded samples
Source: Commun Biol. 2021 Dec 15;4:1396. doi: 10.1038/s42003-021-02930-4 (PMC8674242; doi:10.1038/s42003-021-02930-4)
Supplement: Supplementary file 2 — Supplementary Information [file 42003_2021_2930_MOESM2_ESM.pdf]

Supplementary Information

MicroSEC filters sequence errors for formalin-fixed and paraffin-embedded samples

Masachika Ikegami[1,2,3,*], Shinji Kohsaka[1,*], Takeshi Hirose[1,4], Toshihide Ueno[1], Satoshi Inoue[1], Naoki Kanomata[5], Hideko Yamauchi[6], Taisuke Mori[7], Shigeki Sekine[7], Yoshihiro Inamoto[8], Yasushi Yatabe[7,9], Hiroshi Kobayashi[2], Sakae Tanaka[2], and Hiroyuki Mano[1,*]

[1]Division of Cellular Signaling, National Cancer Center Research Institute, Tokyo, Japan

[2]Department of Orthopaedic Surgery, Faculty of Medicine, The University of Tokyo, Tokyo, Japan

[3]Department of Musculoskeletal Oncology, Tokyo Metropolitan Cancer and Infectious Diseases Center Komagome Hospital, Tokyo, Japan

[4]Department of Orthopaedic Surgery, Graduate School of Medical Sciences, Kyushu University, Fukuoka, Japan

[5]Department of Pathology, St Luke's International Hospital, Tokyo, Japan

[6]Department of Breast Surgical Oncology, St Luke's International Hospital, Tokyo, Japan

[7]Division of Molecular Pathology, National Cancer Center Research Institute, Tokyo, Japan

[8]Department of Hematopoietic Stem Cell Transplantation, National Cancer Center Hospital, Tokyo, Japan

[9]Department of Biobank and Tissue Resources, National Cancer Center Research Institute, Tokyo, Japan

*Correspondence should be addressed to: Masachika Ikegami (ikegami-tky@umin.ac.jp), Shinji Kohsaka (skohsaka@ncc.go.jp), or Hiroyuki Mano (hmano@ncc.go.jp).

Contents

Supplementary figures 1–5

Supplementary tables 1–3

Supplementary Figure 1. The distribution of the variant allele frequencies of the breast tissue samples.

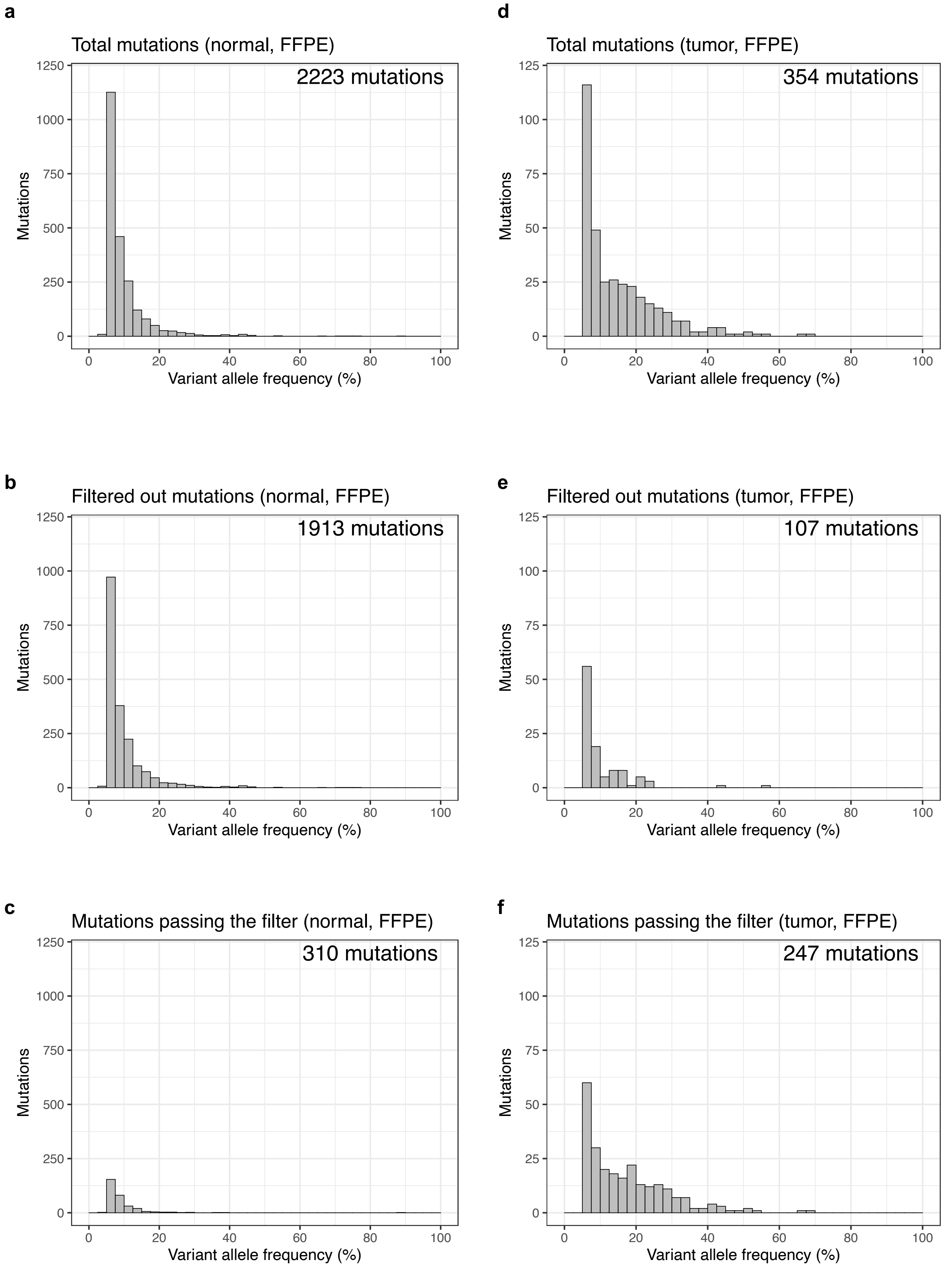

a–c FFPE samples of normal breast tissues (n = 190) with total somatic mutations (**a**), mutations filtered out by MicroSEC filter (**b**), and mutations passing through the filter (**c**).
d–f FFPE samples of breast tumor tissues (n = 33) with total somatic mutations (**d**), mutations filtered out by MicroSEC filter (**e**), and mutations passed through the filter (**f**). The somatic mutations shown represent those present in normal breast tissue but not in normal blood. FFPE, formalin-fixed and paraffin-embedded.

Supplementary Figure 2. The distribution of the mutations in breast tissue samples.

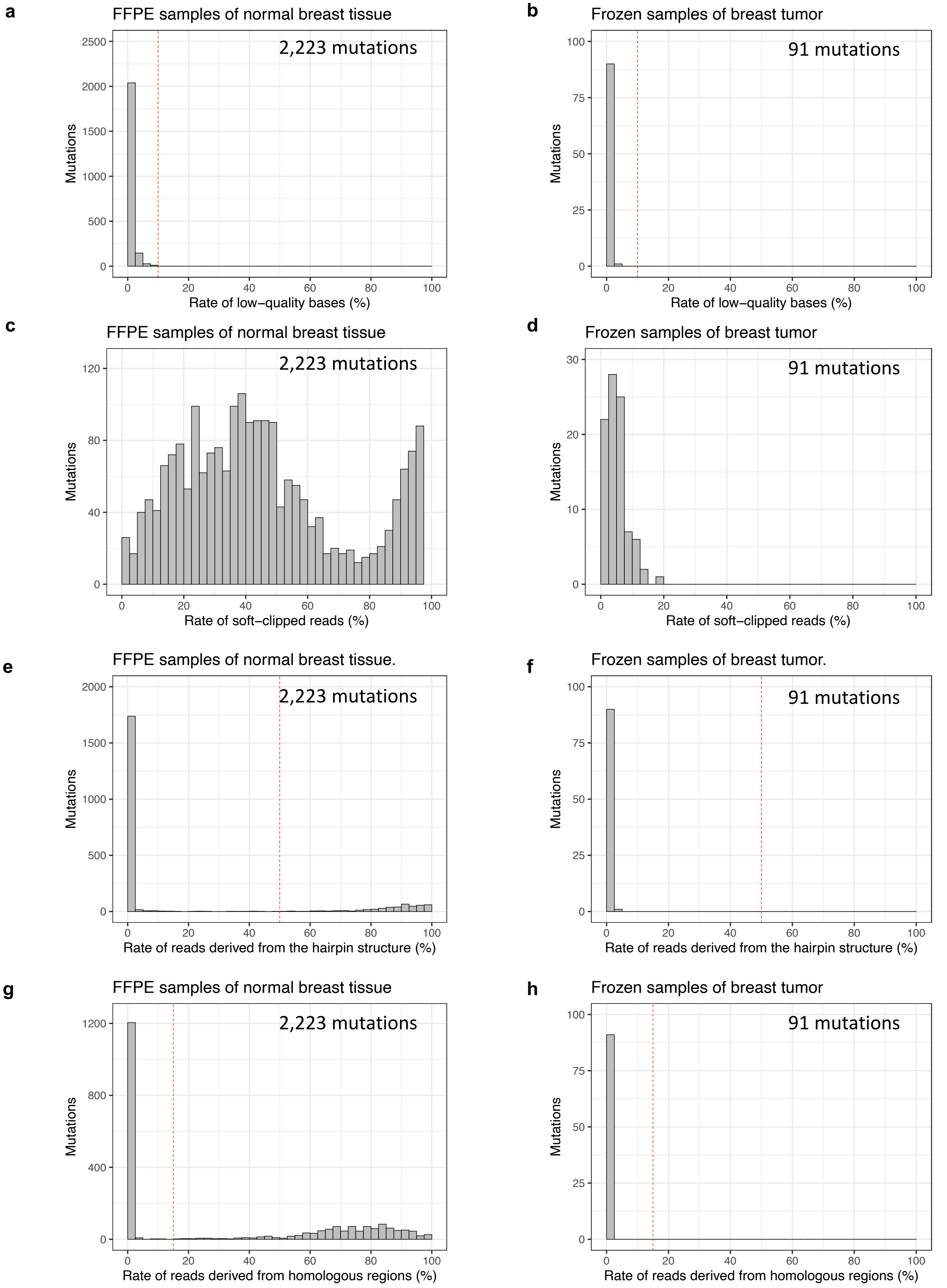

a,b The rate of low-quality bases in mutation-supporting reads in 190 FFPE normal breast tissue samples (**a**) and 23 frozen breast tumor samples (**b**). **c,d** The rate of soft-clipped reads in FFPE samples of normal breast tissue (**c**) and frozen samples of breast tumor (**d**). **e,f** The rate of reads derived from other homologous regions in FFPE samples of normal breast tissue (**e**) and frozen samples of breast tumor (**f**). **g,h** The rate of reads derived from the hairpin structure in FFPE samples of normal breast tissue (**g**) and frozen samples of breast tumor (**h**). Dotted red lines represent the thresholds.

Supplementary Figure 3. The optimal hyperparameters of MicroSEC.

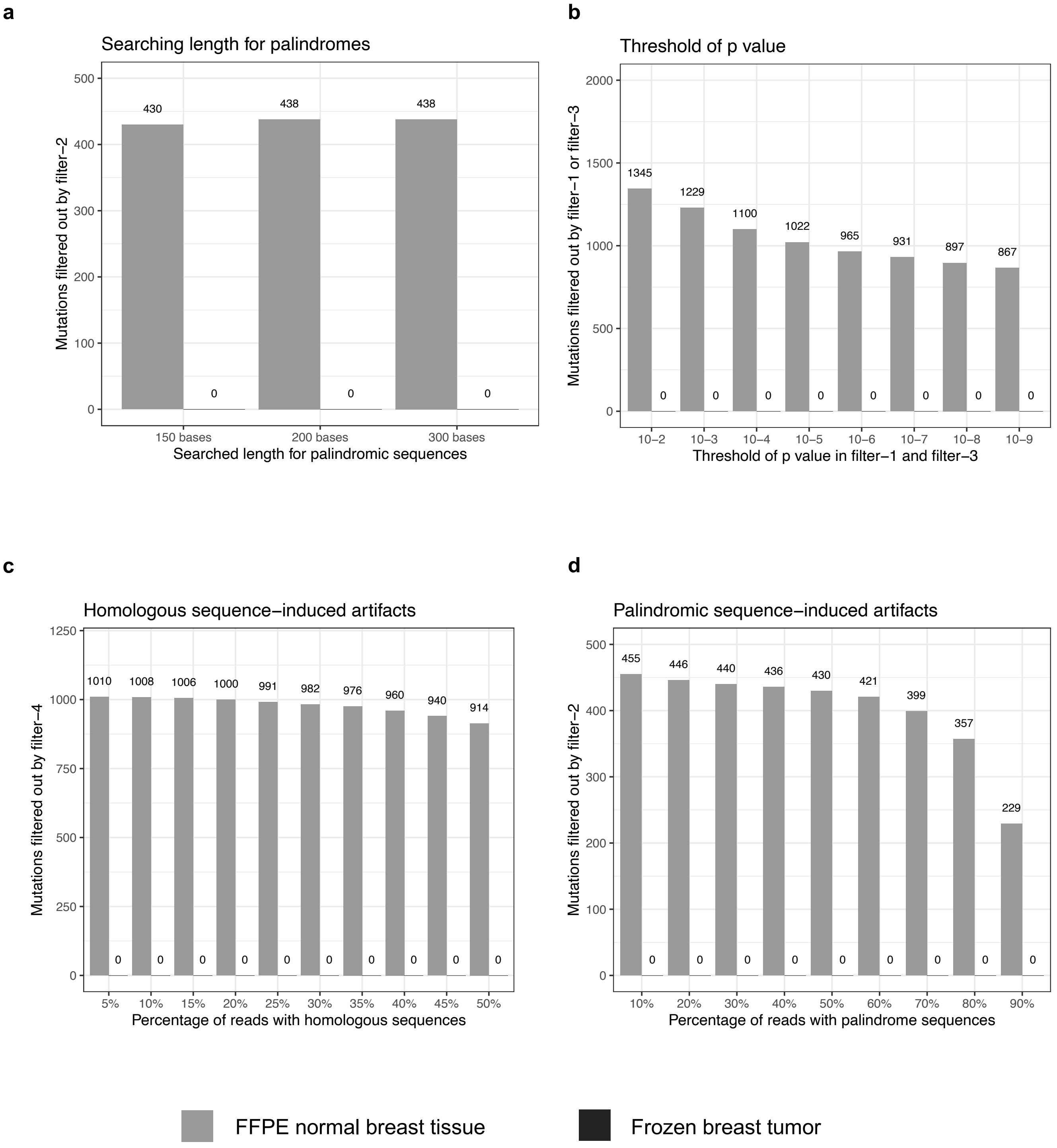

Detected artifacts with various hyperparameters in 190 FFPE normal breast tissue (gray) and 23 frozen breast tumor (black) samples. The base length to search palindromes (**a**), P-value thresholds for Filters 1 and 3 (**b**), Filter 2 (**c**), and Filter 4 (**d**) were varied and the number of artifacts detected was counted. FFPE, formalin-fixed and paraffin-embedded.

Supplementary Figure 4. Aligned reads in capture-based sequencing visualized by Integrative Genomics Viewer.

a *NFYA* p.Gln155Pro, chr6;41090226–41090227delinsCT

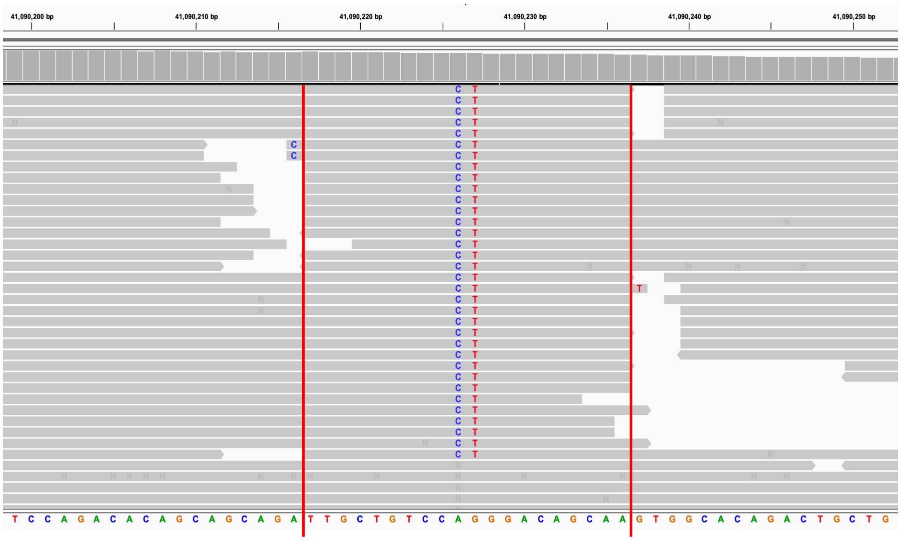

b *CENPA* p.Leu91Pro, chr2;26792817T>C

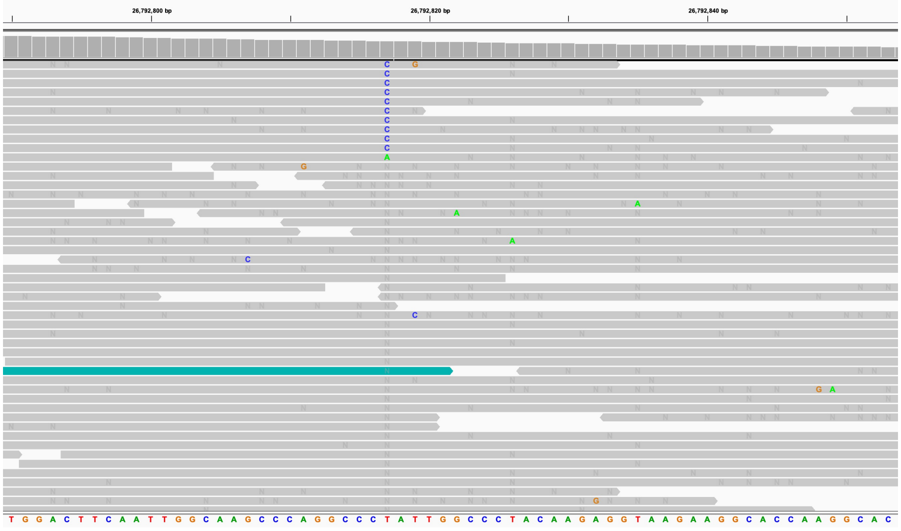

a AG-to-CT mutation in the NFYA gene is shown. All reads with mutations have a short supporting length from the mutated base to the end of the read (red line).
b T-to-C mutation in the CENPA gene is shown. Of the 954 reads mapped to the mutated base, 227 reads (24%) were of low quality and failed to call bases, 689 were wild-type (T), 47 were C, and one was A. Low quality bases are indicated by N. The mate-read of the green colored read is mapped to a different chromosome.

Supplementary Figure 5. Limitation on the number of bases to map around a mutation.

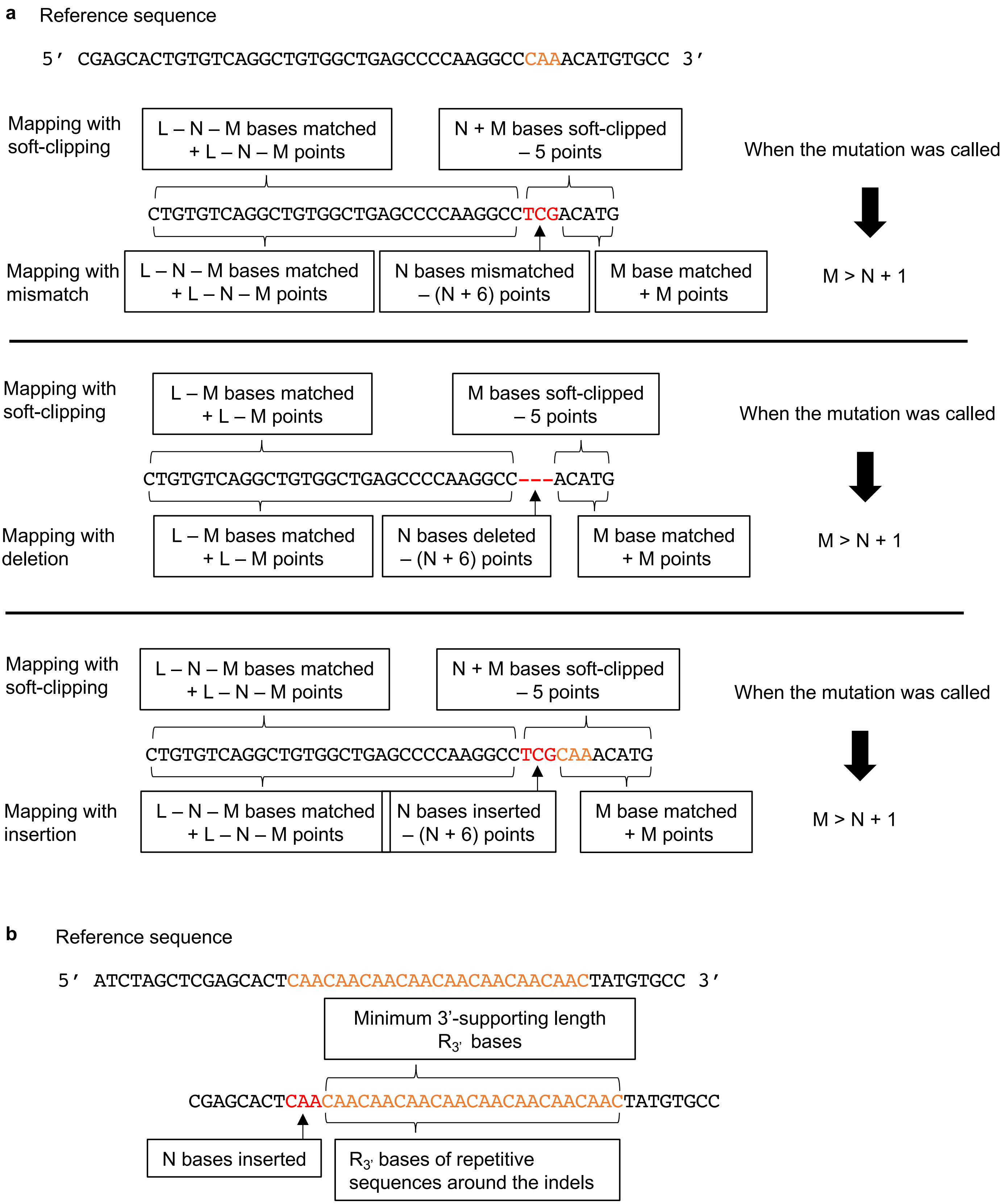

a L was considered to be the read length, N the number of bases mutated, and M the number of bases mapped outside the mutation. When Burrows-Wheeler Aligner were used as a mapper, the penalty due to an N-base mutation was N + 6, the soft-clipping penalty was 5, and the point for mapped M bases was M. When the mutation is called and not soft-clipped, M > N + 1 must be satisfied regardless of the type of mutation.
b If the number of repetitions changes in a short tandem repeat, only reads containing all the repetitive sequences can support the presence of indel mutations.

Supplementary Table 1. Filtered mutations with high variant allele frequency in FFPE samples.

Sample	Chr	Position	Ref	Alt	VAF (%)	Mutation depth	Soft-clipped read	Hairpin-structure	Read length	Supporting length			Probability		
										5’	3’	Shorter	5’	3’	Shorter
PT001_012	12	106255381	G	GTGAC	55.4	655	648 (98.9%)	632 (96.5%)	150	145	125	10	NA	NA	0
PT102_009	12	106255382	A	T	70.2	539	477 (88.5%)	524 (97.2%)	150	148	136	36	NA	NA	NA
PT107_002	12	106255382	A	T	74.3	927	818 (88.2%)	897 (96.8%)	150	149	136	37	NA	NA	NA
PT107_009	12	106255382	A	T	53.4	385	341 (88.6%)	372 (96.6%)	150	147	139	38	NA	NA	NA
PT107_010	12	106255382	A	T	75.9	960	889 (92.6%)	937 (97.6%)	150	149	136	37	NA	NA	NA
PT112_008	12	106255382	A	T	65.4	464	411 (88.6%)	449 (96.8%)	124	123	121	32	NA	NA	NA
PT107_006	12	106255382	A	T	56.7	393	345 (87.8%)	372 (94.7%)	124	123	110	34	NA	NA	NA

Chr, chromosome; Ref, reference sequence; Alt, altered sequence; VAF, variant allele frequency; NA, not assessed.

Probability is calculated only if the supporting length is < 80% of the read length.

Supplementary Table 2. Pathogenic mutations in clinical FFPE samples filtered out by MicroSEC.

Sample	Gene	HGVS.c	HGVS.p	VAF (%)	Mutation depth	Soft-clipped read	Read length	Supporting length			Reads from distant region	MicroSEC
								5’	3’	Shorter		
BRCA_001	*RAD51B*	c.1111C>T	p.Gln371*	12.2	41	26 (63.4%)	150	146	39	39	22 (53.7%)	Filter 4
COAD_001	*PPP2R1A*	c.108delT	p.Leu36fs	13.4	18	5(27.8%)	150	46	141	46	10 (55.6%)	Filter 4
LUAD_016	*FAM175A*	c.229C>T	p.Arg77*	11.8	18	1 (6.3%)	151	141	68	68	12 (66.7%)	Filter 4
LUAD_016	*RAD51B*	c.1111C>T	p.Gln371*	10.9	100	65 (65.0%)	151	150	56	56	42 (42.0%)	Filter 4
LUAD_016	*PPP2R1A*	c.108delT	p.Leu36fs	21.1	25	7 (28.0%)	151	63	145	63	15 (60.0%)	Filter 4
LUAD_021	*TP53*	c.1021T>G	p.Phe341Val	8.8	74	4 (5.4%)	151	102	150	75	0 (0%)	Filter 3
PDC_001	*PPP2R1A*	c.108delT	p.Leu36fs	9.3	28	8 (28.6%)	151	62	144	62	15 (53.6%)	Filter 4
PDC_001	*ZRSR2*	c.283C>T	p.Arg95*	6.0	13	8 (61.5%)	151	149	115	55	7 (53.8%)	Filter 4

Chr, chromosome; Ref, reference sequence; Alt, altered sequence; NA, not assessed.

Probability is calculated only if the supporting length is < 80% of the read length.

Supplementary Table 3. MicroSEC filtering summary for whole exome sequencing.

	Matched primary cancer samples	
	Fresh frozen (N = 14)	FFPE (N = 14)
Total reads (in millions)	111.8 (45.2–145.9)	142.7 (83.6–235.4)
Mapped reads (%)	93.3 (92.8–93.7)	93.4 (85.2–94.1)
Unique reads (%)	86.3 (83.8–93.0)	86.5 (73.5–92.2)
Mean coverage	199 (83–261)	255 (134–394)
Median insert size (base)	223 (197–238)	173 (124–205)
Somatic mutations	107.0 (81–196)	118.2 (94–167)
removed by		
Filter 1	0.1 (0–1)	8.2 (0–47)
Filter 2	0 (0–0)	3.9 (0–23)
Filter 3	0.1 (0–1)	7.3 (0–42)
Filter 4	0.4 (0–3)	1.2 (0–4)
Any of Filter 1–4	0.6 (0–3)	10.3 (0–55)
Mutations passing the filter	106.4 (81–196)	107.9 (85–138)
Filtered rate (%)	0.5	8.7
CG-to-TG potential artifacts	NA	45.8 (14–56)
Intra ≥10-base homopolymer	0.0 (0–0)	0 (0–0)
Remaining mutations	106.4 (81–196)	62.1 (45–89)

Data are shown as mean (range).

NA, not applicable; FFPE, formalin-fixed and paraffin-embedded.
