# Supplementary material for: MicroSEC filters sequence errors for formalin-fixed and paraffin-embedded samples
Source: Commun Biol. 2021 Dec 15;4:1396. doi: 10.1038/s42003-021-02930-4 (PMC8674242; doi:10.1038/s42003-021-02930-4)
Supplement: Supplementary file 3 — Description of Additional Supplementary Files [file 42003_2021_2930_MOESM3_ESM.pdf]

Description of Additional Supplementary Files

File name: Supplementary Data 1.
Description: List of mutations and oligos in this study. Source data underlying Fig. 7 and Supplementary Figs. 1 and 2.

File name: Supplementary Data 2.
Description: Source data underlying Figs. 4, 5b, 6a, and 6b.
