# Supplementary material for: MicroSEC filters sequence errors for formalin-fixed and paraffin-embedded samples
Source: Commun Biol. 2021 Dec 15;4:1396. doi: 10.1038/s42003-021-02930-4 (PMC8674242; doi:10.1038/s42003-021-02930-4)
Supplement: Supplementary file 6 — Reporting Summary [file 42003_2021_2930_MOESM6_ESM.pdf]

nature research

Corresponding author(s): Masachika Ikegami

Last updated by author(s): Nov 16, 2021

Reporting Summary

Nature Research wishes to improve the reproducibility of the work that we publish. This form provides structure for consistency and transparency in reporting. For further information on Nature Research policies, see our Editorial Policies and the Editorial Policy Checklist.

Statistics

For all statistical analyses, confirm that the following items are present in the figure legend, table legend, main text, or Methods section.

n/a	Confirmed	
☐	☒	The exact sample size (*n*) for each experimental group/condition, given as a discrete number and unit of measurement
☐	☒	A statement on whether measurements were taken from distinct samples or whether the same sample was measured repeatedly
☒	☐	The statistical test(s) used AND whether they are one- or two-sided<br>*Only common tests should be described solely by name; describe more complex techniques in the Methods section.*
☒	☐	A description of all covariates tested
☒	☐	A description of any assumptions or corrections, such as tests of normality and adjustment for multiple comparisons
☐	☒	A full description of the statistical parameters including central tendency (e.g. means) or other basic estimates (e.g. regression coefficient) AND variation (e.g. standard deviation) or associated estimates of uncertainty (e.g. confidence intervals)
☒	☐	For null hypothesis testing, the test statistic (e.g. *F*, *t*, *r*) with confidence intervals, effect sizes, degrees of freedom and *P* value noted<br>*Give P values as exact values whenever suitable.*
☒	☐	For Bayesian analysis, information on the choice of priors and Markov chain Monte Carlo settings
☒	☐	For hierarchical and complex designs, identification of the appropriate level for tests and full reporting of outcomes
☒	☐	Estimates of effect sizes (e.g. Cohen's *d*, Pearson's *r*), indicating how they were calculated

Our web collection on statistics for biologists contains articles on many of the points above.

Software and code

Policy information about availability of computer code

Data collection

We performed target deep sequencing of normal breast tissue samples, breast cancer samples, and normal bloodsamples on MiSeq, HiSeq 2500, or NovaSeq6000 (Illumina), or DNBSEQ-G400 (MGI technologies) platform generating 124–151 base-pair paired-end reads.

Data analysis

Sequencing reads were aligned to the reference human genome (hg38) using BWA-MEM and Bowtie2. We used MuTect2 and VarScan2 to call somatic mutations with default settings, and MicroSEC to filter artifacts in FFPE samples. The mapped reads and called mutations were visualized with the Integrative Genomics Viewer. Data management was performed on R language.

Versions of software used:
Bowtie2 v2.1.0
Burrows-Wheeler Aligner v0.7.17
Integrative Genomics Viewer v2.4.10
MicroSEC v1.2.8
MuTect2 GATK v4.1.3.0
R language v4.0.5
SAMtools v1.9
VarScan2 v2.4.3
SOBDetector v1.0.2
ggplot2 v3.3.3
epiR v2.0.29

For manuscripts utilizing custom algorithms or software that are central to the research but not yet described in published literature, software must be made available to editors and reviewers. We strongly encourage code deposition in a community repository (e.g. GitHub). See the Nature Research guidelines for submitting code & software for further information.

Data

Policy information about availability of data

All manuscripts must include a data availability statement. This statement should provide the following information, where applicable:

- Accession codes, unique identifiers, or web links for publicly available datasets
- A list of figures that have associated raw data
- A description of any restrictions on data availability

We have deposited the raw sequencing data of the breast tissue samples and primary cancer samples examined in this study under the accession numbers JGAS000368 and JGAS000377, respectively, in the Japanese Genotype-Phenotype Archive (http://trace.ddbj.nig.ac.jp/jga) hosted by the DNA Data Bank of Japan. The target sequencing data of 54 cancer specimens are available for download at the Japanese Genotype-Phenotype Archive under the accession number JGAS000164.
The raw data for the figures and tables are listed in the Supplementary Data.

Field-specific reporting

Please select the one below that is the best fit for your research. If you are not sure, read the appropriate sections before making your selection.

☒ Life sciences ☐ Behavioural & social sciences ☐ Ecological, evolutionary & environmental sciences

For a reference copy of the document with all sections, see nature.com/documents/nr-reporting-summary-flat.pdf

Life sciences study design

All studies must disclose on these points even when the disclosure is negative.

Sample size	Sample size was not predetermined. Because the sample size was limited by the number of hereditary breast and ovarian cancer syndrome patients and primary cancer patients, we included as many patients as possible in our analysis.
Data exclusions	Samples that did not meet the requirement for sequencing coverage were excluded from this study.<br>We excluded samples with an insufficient mean coverage of less than 400 (n = 28, 8.6%).
Replication	Not applicable. This study uses a filtering algorithm for FFPE artifacts without statistical analysis.
Randomization	Not applicable as no statistical analysis is involved.
Blinding	Not applicable as no statistical analysis or grouping is involved.

Reporting for specific materials, systems and methods

We require information from authors about some types of materials, experimental systems and methods used in many studies. Here, indicate whether each material, system or method listed is relevant to your study. If you are not sure if a list item applies to your research, read the appropriate section before selecting a response.

Materials & experimental systems

n/a	Involved in the study	
☒	☐	Antibodies
☒	☐	Eukaryotic cell lines
☒	☐	Palaeontology and archaeology
☒	☐	Animals and other organisms
☐	☒	Human research participants
☒	☐	Clinical data
☒	☐	Dual use research of concern

Methods

n/a	Involved in the study	
☒	☐	ChIP-seq
☒	☐	Flow cytometry
☒	☐	MRI-based neuroimaging

Human research participants

Policy information about studies involving human research participants

Population characteristics	The study cohort comprised 26 patients with breast cancer who underwent tumor resection or prophylactic surgery because of hereditary breast and ovarian cancer syndrome at St. Luke's International Hospital between 2010 and 2020, and 14 patients with primary cancer (12 colorectal adenocarcinoma and two oral squamous cell carcinoma) resected at National Cancer Center Hospital between 2012 and 2019.

Recruitment	We recruited patients who underwent surgery at St. Luke's International Hospital and had the opportunity to be seen in 2019-2020. Tissue samples of primary cancer were provided by the National Cancer Center Biobank, Japan.
Ethics oversight	The study protocol was approved by the Ethics Committees of St Luke's International Hospital and the National Cancer Center Research Institute. Written informed consent was obtained from all the participants.

Note that full information on the approval of the study protocol must also be provided in the manuscript.
